# BK virus‐induced nephritis and cystitis after matched unrelated donor stem cell transplantation: A case report

**DOI:** 10.1002/ccr3.3246

**Published:** 2020-09-07

**Authors:** Nadine Gelbrich, Matthias B. Stope, Sander Bekeschus, Martin Weigel, Martin Burchardt, Uwe Zimmermann

**Affiliations:** ^1^ Department of Urology University Medicine Greifswald Greifswald Germany; ^2^ Department of Gynaecology and Gynaecological Oncology University Medicine Bonn Bonn Germany; ^3^ ZIK Plasmatis Leibniz Institute for Plasma Science and Technology (INP Greifswald) Greifswald Germany; ^4^ Department of Hematology and Oncology University Medicine Greifswald Greifswald Germany

**Keywords:** acute renal failure, BK virus cystitis, BK virus nephritis, case report, graft‐versus‐host disease, matched unrelated donor stem cell transplantation, myelodysplastic syndromes

## Abstract

Currently, there is no standard therapy for a BK virus infection of the urogenital tract in immunocompromised, stem cell transplanted patients, so that early diagnosis and introduction of supportive measures have the highest response rates to date.

## Graphical abstract

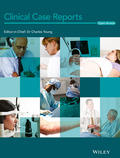

## 1 BACKGROUND

This case report demonstrates the importance of rapid treatment for BK virus infection in immunocompromised patients following matched unrelated donor (MUD) stem cell transplantation.

Currently, very little case data are available, so there is no established standard treatment for a BK virus infection of the urogenital tract. The lack of data on the treatment and prognosis of BK virus infections of the urogenital tract requires long‐term observations that should serve as a basis for future recommendations.

## 2 CASE PRESENTATION

A 39‐year‐old patient presents in November 2018 with myelodysplastic syndrome with excess of blasts (MDS‐EB2). Therefore, the patient was treated with an allogenic stem cell transplantation from a matched unrelated donor in March 2019. Immunosuppression was conducted with cyclosporin A, alemtuzumab, and short‐course methotrexate (MTX). He developed extensive disease chronic graft‐versus‐host disease (GVHD) with mucositis and infestation of the hands, feet, and oral mucosa. Systemic methylprednisolone therapy (60 mg/d) was initiated for treatment.

The initial presentation in July 2019 was acute due to thoracic complaints with dyspnoea, tachycardia, and deterioration of general condition in hyperosmolar hyperglycemia with blood sugar values of 45 mmol/L. Under inpatient conditions, insulin therapy and volume substitution were initiated. The cause of the blood sugar derailment was, eg, a therapy with methylprednisolone in the context of chronic GVHD therapy since June. During the inpatient stay and after completion of the chronic GVHD therapy, the patient increasingly complained of flank pain on the left side with clinical signs of pyelonephritis and macrohematuria. The microbiological examination of the urine revealed a urinary tract infection with Enterococcus faecalis, which is why a test‐appropriate infection therapy with Tazobactam was initiated. In addition, in the hemoglobin‐effective macrohematuria, transfusion‐obligatory anemia was treated with the transfusion of 18 erythrocyte and four platelet concentrates. In case of persistent coagel colics, macrohematuria requiring irrigation and sonographic evidence of a urinary transport disorder II ° left, a CT abdomen was performed natively, excluding a bilateral nephrolithiasis. Due to the persisting clinical situation, a DJ‐catheter was inserted on the left side under test‐appropriate infection therapy with Tazobactam and a transurethral continuous irrigation therapy was initiated. Cystoscopically, there was no evidence of an exophytic tumor. Intravesically an enlarged vascular drawing with signs of cystitis was presented. The left ostium showed tissue necrosis in the orifice and was functionally mute. In addition, blood was withdrawn from the ostium. In the retrograde view, the ureter was inconspicuous up to the level of the sacroiliac joint and the renal pelvis was dull. Bloody urine was also drained from the renal pelvis. An urethral stricture was also found in the area of the fossa navicularis and the pars pendulans.

Under anti‐infectious therapy, DJ‐catheter supply, continuous irrigation therapy with physiological saline solution and diuretic promotion with diuretics, the patient's pain symptoms gradually improved, and the urinary transport disorder on the left and macrohematuria decreased. The cause of the nephritis, as well as the cystitis with a proteinuria of 5.7 g/d and acute renal failure (AKIN 2, RIFLE I), was proven to be an infection with the BK virus (4 480 000 000 copies/mL in urine). The BK virus PCR in the blood was negative so that a BK virus urosepsis could be excluded. In addition, cyclosporin A therapy was reduced in cases of proven tubulo‐interstitial BK virus nephropathy and BK virus nephritis and cidofovir therapy was initiated at reduced dosage (1 mg/kg bw every 2 days) in cases of renal insufficiency. In addition, hypogammaglobulinemia was detected so that the patient received additional human IgG‐preparations (Privigen). Due to the preinterventionally initiated, test‐appropriate infection therapy with Tazobactam, the permanent transurethral catheter and DJ‐catheter were not changed. Under the above measures, flank pain on the left side and macrohematuria decreased, so that transurethral irrigation therapy was discontinued and the DJ‐catheter and the permanent transurethral catheter were removed. Paraclinically, the infection values also decreased with persistently high retention values (creatinine 170 µmol/L), so that the infection therapy was also discontinued. Spontaneous micturition was initially complicated by severe dysuric symptoms (alguria, nocturia) with bloody urine without residual urine formation so that an infection of the proximal urethra with the BK virus could be assumed. After 8 weeks of inpatient treatment, the clinical situation improved and the follow‐up of the urine showed a decrease in the viral load (17 360 BK virus copies in the urine) so that the patient was discharged home with clear urine conditions.

Immunosuppression was tapered from day 43 after stem cell transplantation and discontinued on day 185. At the last follow‐up, he was well and alive, the MDS was in complete remission, and he had no urological symptoms.

## 3 DISCUSSION AND CONCLUSIONS

Myelodysplastic syndromes (MDS) are a heterogeneous group of bone marrow diseases. In Germany, 4.1/100 000 people suffer from myelodysplasia.^1^ As a disease of advanced age with an age median at an initial diagnosis of >65 years, it rarely affects younger patients, as in our case report.^2^ Due to a clonal differentiation disorder of hematopoiesis, the bone marrow of this patient group is no longer able to produce fully mature and functional blood cells. In advanced stages, more and more immature blood cells are produced, so that this hematopoietic process is permanently disturbed. This leads to peripheral cytopenia with the appearance of very immature and nonfunctional blood cells (blasts) in the peripheral blood system and the appearance of acute myeloid leukemia, eg, with excessive blasts.

The cytogenetic alteration is important in this group of diseases and serves, among other things, to stratify the risk. The prognosis varies risk‐factor dependent between some years and few months. Acute leukemias as a consequence of MDS are high‐risk leukemias for which a curative therapeutic approach is currently only available through intensive induction therapy with the aim of MUD stem cell transplantation. Three types of complications after allogeneic stem cell transplantation dominate, which can be life‐limiting. In the phase of aplasia, partially uncontrollable infections with viruses, bacteria, and fungi can occur.^3^ Direct toxic side effects of myeloablative chemotherapy/radiation are inflammation of the mucous membranes/stomatitis, nausea, vomiting, diarrhea, hemorrhagic cystitis, hair loss, and organ‐specific side effects of cytostatic drugs. Serious, sometimes life‐threatening complications are GVHD, which occurs more frequently (up to 50%) in bone marrow transplants than in stem cell transplants. A distinction can be made between acute and chronic forms. Acute GVHD occurs within 3 months after transplantation and usually affects the skin, intestine, and liver, which is why prophylaxis with cyclosporin A and methotrexate (MTX) is performed as a preventive measure. The chronic form of GVHD appears from about 100 days after transplantation and mainly affects the skin and mucous membrane so that immunosuppressive GVHD prophylaxis with glucocorticoids and facultatively other immunosuppressive drugs is necessary.^4^, ^5^ After MUD stem cell transplantation, immunocompromised patients are very susceptible to opportunistic infectious diseases. Thus, these pathogens can remain asymptomatic and become symptomatic before and during immunosuppression, not only in the early neutropenic phase but also after hematopoietic reconstitution due to the persistent T‐ and B‐cell defect.^6^

Viral diseases belong to these opportunistic diseases. The BK virus, a polyomavirus first described in 1970, belongs to the Papovaviridae family and is an ubiquitous, species‐specific virus. Infection with the virus usually occurs in childhood with mild symptoms up to clinically blunt disease progression. Eighty percent of the general population has detectable antibodies against BK viruses, which persist in the body of those affected for life.^7^, ^8^, ^9^ Transmission occurs by smear infection with urine, droplet infection, or contaminated drinking water. In immunocompromised patients, the BK virus can be reactivated or acquired primarily.^9^ BK viruses are involved in several human clinical conditions and are most commonly associated with kidney involvement, such as ureteral stenosis, hemorrhagic cystitis, interstitial nephritis, and nephropathy.^7^, ^8^, ^10^, ^11^ BK nephropathy can lead to gradual deterioration of renal function with an increase in serum creatinine concentration and, in kidney transplant recipients, even loss of the transplant. The tubular epithelial cells are often attacked, which can lead to tubulo‐interstitial nephritis.^7^, ^8^ With significant morbidity, hemorrhagic BK virus cystitis occurs in about 13% (5%‐25%) of patients early after stem cell transplantation.^11^, ^12^, ^13^ Symptoms vary widely from mild dysuria, painless hematuria to painful hematuria or even severe hemorrhagic cystitis with intravesical coagulation and urethral obstruction and possible secondary renal failure.^14^ In bone marrow transplants, the BK virus is often found in urine as part of the virus reactivation process. The polymerase chain reaction (PCR) is used to detect BK viruses in blood, urine, or cerebrospinal fluid. The presence of bait cells in urine and viremia can serve as a noninvasive marker of BKV replication. Immunocompromised individuals may have elevated viral DNA levels even in the absence of clinical signs, so PCR diagnosis is only used to rule out BKV infection. In addition, this marker is also useful in identifying patients at risk of nephropathy and in adjusting immunosuppressive therapy for such patients, as the most effective treatment strategy involves early diagnosis with regular blood and urine screening tests and reduction in immunosuppressive therapy. United Network for Organ Sharing data have shown that the incidence of BK viremia in kidney transplant recipients is approximately 13%, while BK virus‐associated nephropathy occurs in nearly 8% of kidney transplant recipients and can lead to loss of allografts.^15^ To date, there is no specific antiviral standard therapy for the treatment of the BK virus, so that in some cases treatment with various antiviral drugs appears to be successful.^10^, ^16^ The reduced cell‐mediated immune response to the BK virus has been shown to correlate with the progression of the BK virus nephropathy so that the therapeutic approach in the case of significant viremia or histologically confirmed BK virus nephropathy is to reduce immunosuppressive therapy.^15^, ^16^ Mayer et al were able to demonstrate the efficacy of intravesical cidofovir application after allogeneic hematopoietic stem cell transplantation in BK virus cystitis.^17^ Since there is currently no approved antiviral medication for BK virus infection, the intravenous (iv) administration of cidofovir was also investigated. With a response between 47% and 88%, this treatment showed considerable renal toxicity, which is a major problem in stem cell transplantation.^18^, ^19^ Local application of cidofovir (5 mg/kg) intravesically led to an overall response rate of 75%. The reduction in the BK virus replication without relevant systemic toxicities compared to iv application was achieved. In case of failure of local therapy and possible induction of significant renal toxicity, the iv application of cidofovir is still an alternative.^17^, ^20^, ^21^ Recent results of plasma medicine have shown that treatment of tumor cells with cold atmospheric plasma (CAP) not only has antiproliferative and antimicrobial but also has virucidal effects on all biological systems. Thus, both direct (superficial treatment) and indirect (treated rinsing solutions) treatments with CAP could represent a further therapeutic option as well as optimization of the BK virus treatment.^22^, ^23^

In summary, due to poor data availability, there is still no coherent standard therapy. Therefore, further retro‐ and prospective studies to optimize the therapy, to improve patient outcome, and to reduce morbidity seem to be essential. Early diagnosis, initiation of supportive measures, dosage reduction in immunosuppressive therapy, and intravesical cidofovir administration have shown the highest response rates in BK virus therapy to date. Regular follow‐up examinations with evidence of decreasing viral load serve to control the disease and seem to be indispensable.

## CONFLICT OF INTEREST

The authors declare no conflicts of interest or competing interests regarding this case report.

## AUTHOR CONTRIBUTIONS

NG: involved in concept, data collection, data interpretation, drafting article, and critical revision of article. MBS, SB, MB, and UZ: involved in critical revision of article and approval of article. MW: collected the data and involved in critical revision of article.

## ETHICAL APPROVAL

Not applicable/required.
